# The Natchez Tornado

**Published:** 1840-09

**Authors:** 


### THE WESTERN JOURNAL.

No. IX.

LOUISVILLE, SEPTEMBER 1, 1840.

### THE NATCHEZ TORNADO.

We are happy to learn by a letter from Prof. Forshey, late of Jefferson College, Mississippi, that he is engaged in preparing a volume on the Tornado at Natchez. He thinks its phenomena highly instructive to the scientifiic meteorologist, and says they have an important bearing on the Espian Theory. From our knowledge of the Professor's qualifications, and his being on the spot at the time, we anticipate a work of high scientific interest. It will probably appear in October. D.

